# P23 Curing the nail

**DOI:** 10.1093/rap/rkad070.044

**Published:** 2023-09-27

**Authors:** Adaeze Ugwoke, Frances Hall

**Affiliations:** Cambridge University Hospital, Cambridge, United Kingdom; Cambridge University Hospital, Cambridge, United Kingdom

## Abstract

**Introduction:**

Ultraviolet light is a well known trigger for inducing cutaneous flares in people who are predisposed or have been diagnosed with cutaneous lupus, it may have far reaching implications, triggering organ threatening disease

Ultraviolet light could be from various sources including the sun, light bulbs and drying lamps used in nail salons to “cure the nails”.

Curing the nails is a term used in the manicure world where UVA type 2 light is used to facilitate complete and quick drying of the nail polish: these type of UV light have been implicated in the pathogenesis of lupus.

**Case description:**

This is the case of a 41 year old lady of African descent diagnosed with discoid lupus at the age of 33. Symptoms include hair loss, hypopigmented lesions on her ear, urticarial rash, fatigue, joint stiffness. Prior history of pregnancy loss at 14 weeks noted, and her sister also suffers from lupus.

On examination she was found to have scarring alopecia with hyperkeratotic erythematous lesions at the periungual area with acrylic nails.

Blood work showed lymphopenia, positive ANA, positive dsDNA by ELISA, RO+, positive anti smith antibody, normal complements and no evidence of kidney involvement.

She was treated with topical therapy and then escalated in turns to hydroxychloroquine, mycophenolate mofetil, azathioprine, rituximab and methotrexate. She had developed a rash on hydroxychloroquine leading to it's discontinuation.

The periungual lesions were resistant to all of the above treatment including intralesional steroid. She had recurrent flares of the lesions which resulted in episodes of infection, pus drainage, and treatment with antibiotics.

Nifedipine was introduced but did not ameliorate symptoms

A causal link was eventually made between her exposure to UV light at the nail salon and the periungual lesion.

She was advised to discontinue using the UV light and possibly resort to traditional nail polish and air drying.

The periungual lesions significantly improved on cessation of using the UV lights.

**Discussion:**

Gel/acrylic manicures are becoming increasingly popular as they are more aesthetically pleasing and long lasting compared to traditional nail polish which uses air drying method, however we should be aware of the risk of ultraviolet (UV) light exposure which is used to dry the nails. UV light stimulates keratinocytes to express more small nuclear ribonucleoproteins thereby secreting IL-1,1L-6, IL-3, GM-CSF which lead to tissue damage and increased autoantibody production, this can result in development of cutaneous lupus, worsening of cutaneous lupus or in some cases can precipitate organ threatening systemic lupus.

As clinicians we should be aware of the risks and educate our patients on the implication to their disease, they should be encouraged not to use this method of manicure but rather resort to the traditional nail polish which uses air drying technique rather than UV light.

Other options may include applying sun protection cream to the fingers before manicure or the use of UV protective gloves, these measures though may not provide adequate coverage to the periungual area.

We should be aware and educate our patients on avoidance of other sources of UV light including avoidance of fluorescent light, tanning beds, surgical lamps and measures to reduce exposure.

**Key learning points:**

Consider other sources of UV light exposure in patients with hard to treat skin disease. It is critical that we educate our patients on the possible side effects of UV exposure and counsel on strategies to minimise exposure

